# Notices

**Published:** 1868-09

**Authors:** 


### NOTICES.

"*American Eclectic Medical Review*," edited by Drs. Robert S. Newton and P. Albert Morrow, published monthly by Trow & Smith, 50 Green Street, New York, $2 a year, single numbers 25 cts. A very instructive article on Life Assurance by John Jamieson M. D. is found in the August number and is well worth the attentive perusal of every husband and father. Sun stroke, Weather and Health. Dr Alexander Wilder advocates Eclecticism and the practice of medicine by women. The Doctor has a charming, intellectual and very handsome wife, would he like for her to spend her days and nights in going round and giving pills to people? It would be a very good plan to educate women to be professional nurses, to be followed until they get married and then to be "retained" exclusively to the family practice for the husband and children. Two benefits would follow, there would be fewer old maids and the average of human life would be largely increased, because every benedict would marry his doctor out of pure gratitude for his restoration and good nursing would save a thousand where physic saves one; not that medicine is less valuable but that scientific nursing is more.

*Welcomed Return.* The Philadelphia City Item very justly says, Our energetic enterprising, and accomplished friend, Mr. J. S. Redfield, returns to the publishing business. Everybody remembers Redfield. A few years since he was among the most prominent publishers in the United States, a position acquired by hard work, honesty, enterprise, liberality, and thorough knowledge of his profession. Mr. Redfield is a gentleman of ample means, his generosity is proverbial, and socially, he is "the best of good fellows." By his exquisite publications he has conferred upon the literary world obligations that cannot be repaid. He gave to the world Darley's exquisite work, "Margaret"—a work unsurpassed, perhaps unequaled throughout the world. This one act, if he had done no more, would endear him to the lovers of classic art-literature—but, he has done much more—he has given us several editions of Shakespeare, besides various works of great and permanent value—Dr. Doran's works, Noctes Ambrosiana, and many others, which were eagerly bought and highly appreciated by a critical and exacting public. We cordially greet the return of Mr. Redfield to a profession which he will adorn

*The Medical Record*, a semi-monthly journal of Medicine and Surgery, edited by George F. Shrady M. D. and published at $4. a year by W. Wood & Co, 61 Walker Street. New York. The number for Aug. 1st, is filled with a great variety of practical and scientific items. We commend it to the Profession.

The Druggists' Circular and Chemical Gazette, published monthly in N.Y. city certainly merits the patronage of the entire medical profession for the variety of its contents in reference to the value and uses of remedies, of new preparations and the latest medical and surgical intelligence throughout the World. L. V. Newton M.D. Editor and Publisher, 36 Beekman Street.

"*From the Oak to the Olive*," being a plain record of a pleasant journey by Julia Ward Howe, Pub'd by Lee & Shepherd Boston, 304 pp 12mo. It is the "record of a journey crowded with interests and pleasures," being the third foreign tour of an accomplished woman who wields a lively chatty pen which turns off sentences with wonderful facility and which are freighted with such a wealth of suggestion and information and enlivenment as we have not noticed in any book of foreign travel from a lady's hand, even. There is a halo of the angelic which surrounds the word "Lady," whether uttered or printed, in the mind of a true knight and gentleman, we the editor for example, but we must confess to a little "falling off on reading that on arriving at "Liverpool a good deal of time was spent in getting something to eat." We have not been in the habit of associating angels heavenly or earthly with vulgar bread and butter, with perhaps, molasses added, but bating that this is one of the most readable books on foreign travel yet issued, not only for those who have been abroad but for those who have that world of happiness in contemplation; it keeps you smiling, joyous and jubilant from first to "Finis."

"Old New York" or reminiscences of the past sixty years, by John M Francis M. D. L. L. D, with a memoir of the author by Henery T. Tuckerman, being one of the many valuable and standard publications of Widdleton, number 27 Howard Street, NewYork, 400 pp.8vo. with an admirable steel engraving of the author, which, by the way strikingly reminds us of the face of our old preceptor Dr B. W. Dudley still living beyond the "fourscore," who not only in his prime possessed one of the greatest surgical minds of the age but whose general ideas of hygiene were so truthful and wise that they find their exemplification in a vigorous and healthful old age. There is not a Knickerbocker family in New York, not a man to the manor born who does not do himself a discredit, if he has not this most interesting book in his library or on his parlor table, giving as it does a personal sketch or family history of very many old New Yorkers and characters of eminence at home and abroad with whom Dr. Francis from his talents and social qualities and influence was thrown. Striking anecdotes of the men of his time are numerous and are intensely interesting, amusing and instructive. Dr. Francis was born in the year of Washington's inauguration as First President of the Republic and died in the year of the "Great Conflict" began between the North and the South, 1789—1861. His last words, as if contemplating his own dissolution, the ruling passion strong in death, were, "I am gone," and so passed away in entire consciousness and without a struggle.

The New-York Medical College for Women issues its sixth annual announcement, together with its charter. Mrs. Wm. H. Grenough, President; Anna M. Innman M.D. Lecturer on Obstetrics and Diseases of Women; and Miss C. S. Lozier M. D. Emeritus Professor of diseases of Women, Dean. Seven gentlemen of eminence and marked ability fill the other chairs of the institution. Miss Emily Schetler was the first and only graduate in the class organized in 1863. There were fourteen graduates the second year, three the third, nine the fourth, and eight the fifth; its requisitions are the same with other medical colleges. Terms and fees $125.00 a session. Donations of books, maps, and money are solicited. Our present opinion is that the more women are exposed to public gaze and contact the more they loose as to the sacredness and elevation of their character; at the same time as the world increases in population, the necessity that women should do something towards supporting themselves when maiden or widowed increases, and it would seem that when they have not the stronger arm of father or husband to lean upon, it is a simple act of justice and right that all the avenues of self-support should be opened to them for which they are capable and adapted, and that several women physicians in New York city return incomes from two to twenty thousand dollars is clear evidence of their fitness (in public estimation) and ability for the performance of the duties of physician, surgeon and obstetrician. But the "wife," sacred name! let her remain the divinity of the household, as long as time endures.

EPILEPSY. The medical profession, and the general reader as well, would do well to read a most valuable and practical editorial article, on the treatment of Epilepsy, in the August number of Cornell's Guardian of Health, 1654 Washington St. Boston. 25cts., post-paid.

Parents of New York and elsewhere will find at the establishment of BALDWIN the Clothier, corner of Broadway and Canal street, a large assortment of boys' clothing, as well as men's which for their quality, are guarranteed to be the cheapest in the city; but to make this a paying institution, with such small profits, the terms are "ONE PRICE, C. O. D."

